# Mental health and social isolation under repeated mild lockdowns in Japan

**DOI:** 10.1038/s41598-022-12420-0

**Published:** 2022-05-19

**Authors:** Tetsuya Yamamoto, Chigusa Uchiumi, Naho Suzuki, Nagisa Sugaya, Eric Murillo-Rodriguez, Sérgio Machado, Claudio Imperatori, Henning Budde

**Affiliations:** 1 Graduate School of Technology, Industrial and Social Sciences, Tokushima University, Tokushima, Japan; 2 Intercontinental Neuroscience Research Group, Tokushima, Japan; 3 Graduate School of Sciences and Technology for Innovation, Tokushima University, Tokushima, Japan; 4 Unit of Public Health and Preventive Medicine, School of Medicine, Yokohama City University, Yokohama, Japan; 5 Laboratorio de Neurociencias Moleculares e Integrativas, Escuela de Medicina, División Ciencias de la Salud, Universidad Anáhuac Mayab, Mérida, Yucatán Mexico; 6 Department of Sports Methods and Techniques, Federal University of Santa Maria, Santa Maria, Brazil; 7 Laboratory of Physical Activity Neuroscience, Neurodiversity Institute, Queimados, RJ Brazil; 8 Cognitive and Clinical Psychology Laboratory, Department of Human Sciences, European University of Rome, Rome, Italy; 9 Institute for Systems Medicine (ISM), Faculty of Human Sciences, Medical School Hamburg (MSH), Hamburg, Germany

**Keywords:** Health care, Public health, Epidemiology, Psychology

## Abstract

The influence of repeated lockdowns on mental health and social isolation is unknown. We conducted a longitudinal study of the influence of repeated mild lockdowns during two emergency declarations in Japan, in May 2020 and February 2021. The analyses included 7893 people who participated in all online surveys. During repeated mild lockdowns, mental and physical symptoms decreased overall, while loneliness increased and social networks decreased. Subgroup analyses revealed that depression and suicidal ideation did not decrease only in the younger age group (aged 18–29 years) and that younger and middle-aged people (aged 18–49 years), women, people with a history of treatment for mental illness, and people who were socially disadvantaged in terms of income had higher levels of mental and physical symptoms at all survey times. Additionally, comprehensive extraction of the interaction structure between depression, demographic attributes, and psychosocial variables indicated that loneliness and social networks were most closely associated with depression. These results indicate that repeated lockdowns have cumulative negative effects on social isolation and loneliness and that susceptible populations, such as young people and those with high levels of loneliness, require special consideration during repeated lockdown situations.

## Introduction

The onslaught of the coronavirus disease 2019 (COVID-19) remains severe as of June 2021^1^. Many countries have implemented lockdowns to prevent the spread of infection. While these lockdowns have been effective in reducing the spread of infection, many negative psychological effects have been reported, including a high prevalence of psychiatric symptoms, such as depression and anxiety^2–7^.

Previous studies reporting the negative effects during lockdown have been limited to cross-sectional surveys^3,4,6^ and follow-up studies under single lockdown situations^5,7^. There have been no longitudinal data demonstrating the effects of repeated lockdowns. Therefore, the question of whether multiple lockdowns have a negative influence on symptoms or whether the influence can be reduced as people become accustomed to lockdown circumstances remains unanswered^8^. Considering that lockdowns have a significant impact on people's lives and economic activities^9^, it can be assumed that repeated lockdowns have different effects on people's mental health and interpersonal interaction styles than single lockdowns. Since repeated lockdown policies have already been implemented in many parts of the world and may continue to be implemented in the future due to new outbreaks, it is essential to elucidate the influence of repeated lockdowns on mental health and interpersonal interactions.

**Figure 1:**
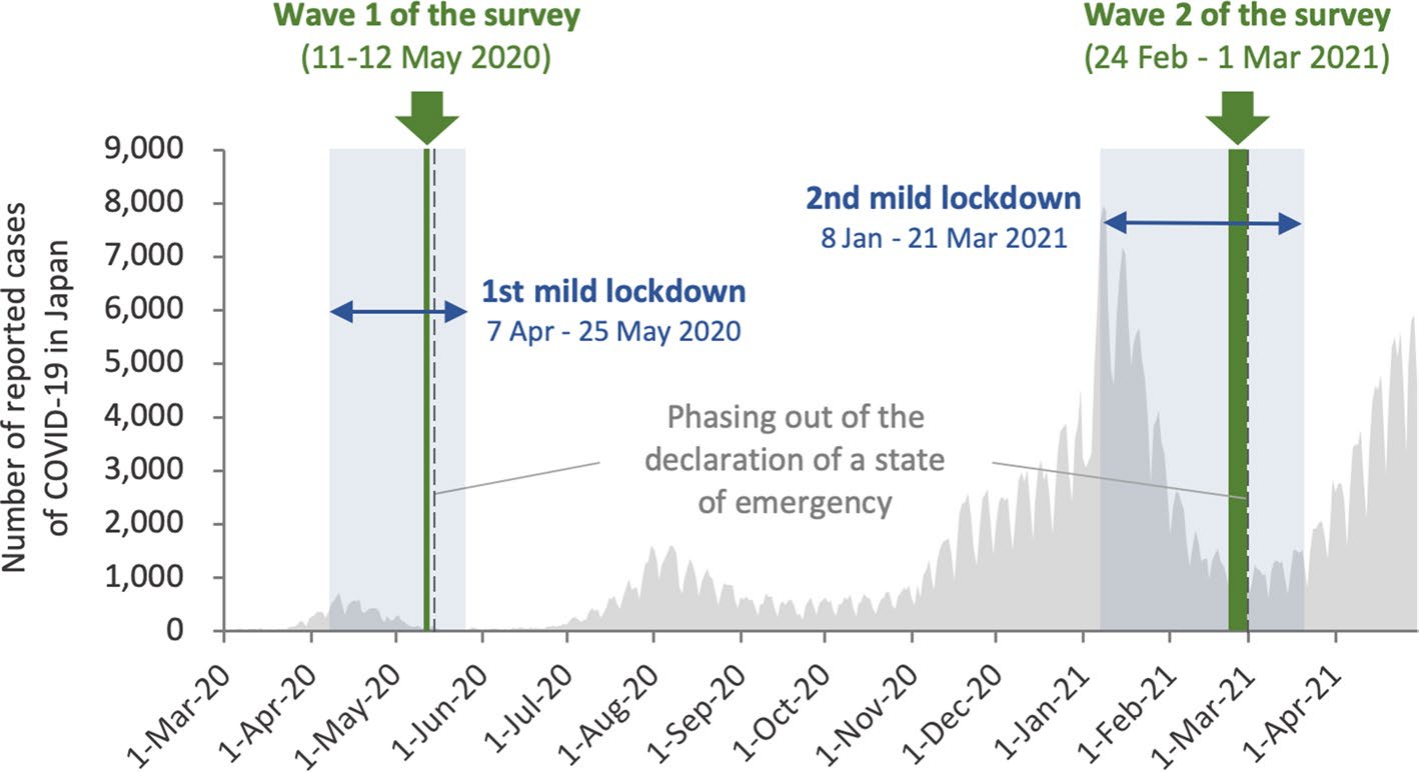
Status of COVID-19 infection in Japan from March 2020 to April 2021 and the study timeline.

Japan is one country that has implemented repeated lockdowns. The Japanese government declared a state of emergency on 7 April 2020 and 8 January 2021 and implemented a "mild lockdown”^6^, an unenforceable request for self-restraint (Fig. 1). During the first mild lockdown, a significant increase in psychological distress and depression was reported despite the lack of legal enforcement of self-restraint^6,10,11^, while the influence of the second mild lockdown on those participants is unclear.

In this lockdown context in Japan, longitudinal studies of mental health characteristics are critical to understanding the influence of repeated lockdowns. Such analyses will provide important fundamental data for improving lockdown approaches that still permit maximum infection prevention and maintain mental health.

The influence of lockdown is also particularly prevalent among certain groups, such as women, the young, the socially disadvantaged in terms of income, and those with a history of mental illness^6,12,13^. Because the effects of repeated lockdowns may be specific to these groups, a detailed examination of these associations would provide information regarding what groups require particular attention.

In this study, we examined the influence of repeated lockdowns on mental health and social isolation by conducting a large-scale panel survey of people in urban areas under two lockdowns due to declared states of emergency in Japan.

## Results

### Participant characteristics

The mean age of those who participated in both surveys (N = 7893, response rate 69.6%, Table 1) was 49.6 years (SD = 13.7), with the 30–49 years age group being the most highly represented (41.8%). Of the respondents, 65.6% were married, and 68.2% were employed. Compared to those who participated in the first wave only (N = 3440), those who participated in both were older and had a larger proportion of men and married people (all p-values < 0.001; Supplementary Table S1). In addition, those who participated in both experienced greater loneliness and smaller social networks, while fewer people exceeded the cut-off points for depression (Patient Health Questionnaire-9 [PHQ9] ≥ 10) and psychological stress (Kessler Psychological Distress Scale [K6] ≥ 5) and reported suicidal ideation in the first survey (all p-values < 0.001; Supplementary Table S1).

**Table 1:** Demographic characteristics of the participants.

Characteristics (N = 7893)	n (%)
**Age, years**	
18–29	637 (8.1%)
30–49	3302 (41.8%)
50–64	2701 (34.2%)
≥ 65	1253 (15.9%)
**Gender**	
Men	4201 (53.2%)
Women	3692 (46.8%)
**Occupation category**	
Employed	5384 (68.2%)
Homemaker	1236 (15.7%)
Student	111 (1.4%)
Unemployed	901 (11.4%)
Other	261 (3.3%)
**Marital status**	
Married	5174 (65.6%)
Unmarried	2719 (34.4%)
**Annual household income (JPY)**	
< 2 million^a^	438 (5.5%)
2 million to < 4 million	1421 (18.0%)
4 million to < 6 million	1562 (19.8%)
6 million to < 8 million	1078 (13.7%)
≥ 8 million	1613 (20.4%)
Unknown	1781 (22.6%)
**Currently receiving treatment for mental illness**	
Yes	431 (5.5%)
No	7462 (94.5%)
**Received treatment for mental illness in the past**	
Yes	875 (11.1%)
No	7018 (88.9%)

^a^2 million JPY = approximately £15,000.

### Differences in living conditions and self-restraint between the first and second waves

Compared to the first wave, the frequency of experiencing household financial difficulties, living difficulties, and academic or occupational difficulties in the second wave decreased significantly (all p-values < 0.001; Supplementary Table S2). In addition, compared to the first wave, consumption activity index improved (wave 1: 82.7; wave 2: 94.4; Supplementary Fig. S1) and public transportation use tended to increase in the second wave (wave 1: − 43 to − 42%; wave 2: − 29 to − 25%; Supplementary Fig. S2).

### Mental health under repeated mild lockdowns in the whole sample

Depression, suicidal ideation, psychological distress, and physical symptoms were significantly lower in the second than the first wave (all p-values < 0.001; Table 2). Alternatively, in the second wave, loneliness increased significantly (b = 0.24, 95% confidence interval [CI] 0.15 to 0.33, p < 0.001) and social network decreased significantly (b =  − 0.43, 95% CI − 0.54 to − 0.33, p < 0.001). The percentage of patients who exceeded the cut-off score for Generalised Anxiety Disorder scale-7 (GAD), measured only in the second wave, was 8.5% (Table 2).

**Table 2:** Changes in outcome variables over waves 1 and 2 with odds ratios, betas, and 95% confidence interval.

N = 7893	Wave 1	Wave 2	OR^a^	95% Cl	p
Depression (PHQ9 ≥ 10), n (%)	1286 (16.3%)	989 (12.5%)	0.74	0.69 to 0.79	< 0.001
Suicidal ideation, n (%)	1503 (19.0%)	1212 (15.4%)	0.77	0.73 to 0.82	< 0.001
Psychological distress (K6 ≥ 5), n (%)	3595 (45.5%)	2507 (31.8%)	0.56	0.53 to 0.58	< 0.001

	Wave 1	Wave 2	Beta (SE)	95% Cl	p
Somatic symptoms, mean (SD)	6.39 (5.50)	4.89 (5.38)	− 1.49 (0.05)	− 1.59 to − 1.40	< 0.001
Loneliness, mean (SD)	23.59 (5.74)	23.83 (5.85)	0.24 (0.05)	0.15 to 0.33	< 0.001
Social network, mean (SD)	10.14 (6.13)	9.71 (6.06)	− 0.43 (0.05)	− 0.54 to − 0.33	< 0.001
GAD7 (% ≥ 10)	–	667 (8.5%)	–	–	–
PHQ9 score, mean (SD)	4.54 (5.40)	3.78 (5.39)	–	–	–
K6 score, mean (SD)	5.27 (5.34)	3.85 (5.11)	–	–	–
GAD7 score, mean (SD)	–	2.84 (4.27)	–	–	–

^a^Reference group: wave 1.

*CI* confidence interval; *OR* odds ratio.

### Mental health under repeated mild lockdowns with a focus on subgroups

The results of the interactions are mainly described, and details of all analyses are provided in Supplement A in the Supplementary Materials.

### Estimated rate of depression

Both men and women displayed a reduction in depression rate in the second wave (Table 3). However, this reduction was significantly smaller among women than men (b = 0.18, 95% CI 0.52 to 0.32, p < 0.001). Women also had a significantly higher estimated rate of depression in both the first and second waves than men (wave 1: odds ratio [OR] = 1.30, 95% CI 1.15 to 1.46, p < 0.001; wave 2: OR = 1.56, 95% CI 1.36 to 1.78, p < 0.001).

**Table 3:** Changes in outcome variables by age group and gender.

	Wave 1					Wave 2				
	18–29 yrs	30–49 yrs	50–64 yrs	≥ 65 yrs	Total	18–29 yrs	30–49 yrs	50–64 yrs	≥ 65 yrs	Total
**Men (n = 4201)**										
Depression (PHQ9 ≥ 10), n (%)	25 (18.2%)	295 (20.7%)	242 (14.0%)	53 (5.8%)	615 (14.6%)	34 (24.8%)	224 (15.7%)	148 (8.6%)	25 (2.7%)	431 (10.3%)
Suicidal ideation, n (%)	32 (23.4%)	366 (25.7%)	333 (19.3%)	71 (7.8%)	802 (19.1%)	31 (22.6%)	293 (20.6%)	219 (12.7%)	36 (3.9%)	579 (13.8%)
Psychological distress (K6 ≥ 5), n (%)	80 (58.4%)	731 (51.4%)	671 (38.9%)	218 (23.9%)	1700 (40.5%)	63 (46.0%)	525 (36.9%)	416 (24.1%)	112 (12.3%)	1116 (26.6%)
Somatic symptoms, mean (SD)	5.28 (5.18)	6.46 (5.76)	6.07 (5.50)	5.17 (4.50)	5.98 (5.40)	4.26 (4.94)	5.20 (5.69)	4.50 (5.31)	3.18 (3.59)	4.44 (5.17)
Loneliness, mean (SD)	24.04 (5.94)	25.16 (5.47)	24.11 (5.39)	21.70 (5.30)	23.94 (5.56)	24.34 (5.37)	25.29 (5.69)	24.12 (5.53)	21.83 (5.40)	24.03 (5.69)
Social network, mean (SD)	11.72 (6.58)	9.04 (6.09)	9.02 (6.18)	10.80 (6.34)	9.50 (6.26)	11.03 (6.34)	8.69 (5.94)	8.67 (6.23)	10.60 (6.25)	9.17 (6.20)
Anxiety (GAD7 ≥ 10), n (%)	–	–	–	–	–	23 (16.8%)	158 (11.1%)	104 (6.0%)	16 (1.8%)	301 (7.2%)
PHQ9 score, mean (SD)	4.99 (5.43)	5.09 (5.69)	4.05 (5.27)	2.32 (3.58)	4.06 (5.21)	5.48 (6.21)	4.42 (5.84)	2.96 (5.15)	1.49 (3.17)	3.22 (5.22)
K6 score, mean (SD)	6.62 (5.67)	5.78 (5.48)	4.46 (4.96)	2.80 (3.48)	4.62 (5.03)	5.84 (6.35)	4.41 (5.48)	2.94 (4.49)	1.56 (2.84)	3.23 (4.78)
GAD7 score, mean (SD)	–	–	–	–	–	3.72 (4.87)	3.42 (4.72)	2.19 (3.94)	1.01 (2.39)	2.40 (4.10)
**Women (n = 3692)**										
Depression (PHQ9 ≥ 10), n (%)	103 (20.6%)	383 (20.4%)	173 (17.8%)	12 (3.5%)	671 (18.2%)	90 (18.0%)	343 (18.3%)	114 (11.7%)	11 (3.2%)	558 (15.1%)
Suicidal ideation, n (%)	112 (22.4%)	389 (20.7%)	174 (17.9%)	26 (7.7%)	701 (19.0%)	112 (22.4%)	370 (19.7%)	134 (13.8%)	17 (5.0%)	633 (17.1%)
Psychological distress (K6 ≥ 5), n (%)	282 (56.4%)	1010 (53.8%)	482 (49.5%)	121 (35.7%)	1895 (51.3%)	234 (46.8%)	774 (41.2%)	325 (33.4%)	58 (17.1%)	1391 (37.7%)
Somatic symptoms, mean (SD)	6.63 (5.40)	7.09 (5.75)	7.03 (5.60)	5.30 (4.48)	6.85 (5.58)	5.52 (5.69)	5.72 (5.71)	5.45 (5.62)	3.35 (3.85)	5.41 (5.58)
Loneliness, mean (SD)	23.44 (5.85)	23.88 (5.92)	22.90 (5.76)	19.72 (5.15)	23.18 (5.92)	24.06 (5.90)	24.29 (6.06)	23.30 (5.80)	19.95 (5.15)	23.60 (6.02)
Social network, mean (SD)	11.39 (5.90)	10.58 (5.83)	10.51 (5.88)	12.60 (5.99)	10.86 (5.90)	10.78 (5.80)	9.97 (5.80)	9.97 (5.79)	12.52 (5.77)	10.31 (5.83)
Anxiety (GAD7 ≥ 10), n (%)	–	–	–	–	–	59 (11.8%)	222 (11.8%)	79 (8.1%)	6 (1.8%)	366 (9.9%)
PHQ9 score, mean (SD)	5.91 (5.62)	5.42 (5.67)	4.88 (5.61)	2.56 (3.55)	5.08 (5.55)	5.51 (5.60)	4.97 (5.77)	3.78 (5.24)	1.63 (2.93)	4.42 (5.51)
K6 score, mean (SD)	6.64 (5.69)	6.41 (5.77)	5.79 (5.44)	3.68 (3.75)	6.02 (5.57)	5.52 (5.72)	4.97 (5.56)	4.10 (5.18)	2.01 (3.15)	4.54 (5.38)
GAD7 score, mean (SD)	–	–	–	–	–	3.86 (4.57)	3.82 (4.64)	2.85 (4.16)	1.33 (2.38)	3.34 (4.41)
**All (N = 7893)**										
Depression (PHQ9 ≥ 10), n (%)	128 (20.1%)	678 (20.5%)	415 (15.4%)	65 (5.2%)	1286 (16.3%)	124 (19.5%)	567 (17.2%)	262 (9.7%)	36 (2.9%)	989 (12.5%)
Suicidal ideation, n (%)	144 (22.6%)	755 (22.9%)	507 (18.8%)	97 (7.7%)	1503 (19.0%)	143 (22.4%)	663 (20.1%)	353 (13.1%)	53 (4.2%)	1212 (15.4%)
Psychological distress (K6 ≥ 5), n (%)	362 (56.8%)	1741 (52.7%)	1153 (42.7%)	339 (27.1%)	3595 (45.5%)	297 (46.6%)	1299 (39.3%)	741 (27.4%)	170 (13.6%)	2507 (31.8%)
Somatic symptoms, mean (SD)	6.34 (5.38)	6.82 (5.76)	6.41 (5.55)	5.20 (4.50)	6.39 (5.50)	5.25 (5.56)	5.50 (5.71)	4.85 (5.44)	3.23 (3.66)	4.89 (5.38)
Loneliness, mean (SD)	23.59 (5.74)	24.43 (5.77)	23.68 (5.56)	21.17 (5.33)	23.59 (5.74)	24.12 (5.79)	24.72 (5.93)	23.83 (5.64)	21.32 (5.39)	23.83 (5.85)
Social network, mean (SD)	11.46 (6.05)	9.92 (5.99)	9.55 (6.11)	11.29 (6.32)	10.14 (6.13)	10.83 (5.87)	9.42 (5.89)	9.14 (6.11)	11.12 (6.18)	9.71 (6.06)
Anxiety (GAD7 ≥ 10), n (%)	–	–	–	–	–	82 (12.9%)	380 (11.5%)	183 (6.8%)	22 (1.8%)	667 (8.5%)
PHQ9 score, mean (SD)	5.71 (5.58)	5.28 (5.68)	4.35 (5.41)	2.38 (3.57)	4.54 (5.40)	5.50 (5.73)	4.73 (5.81)	3.26 (5.20)	1.53 (3.11)	3.78 (5.39)
K6 score, mean (SD)	6.64 (5.68)	6.14 (5.65)	4.94 (5.18)	3.04 (3.58)	5.27 (5.34)	5.59 (5.86)	4.73 (5.53)	3.36 (4.78)	1.68 (2.93)	3.85 (5.11)
GAD7 score, mean (SD)	–	–	–	–	–	3.83 (4.63)	3.65 (4.68)	2.43 (4.04)	1.09 (2.39)	2.84 (4.27)

*PHQ9* Patient Health Questionnaire-9, *K6* Kessler Psychological Distress Scale-6, *GAD7* Generalized Anxiety Disorder Scale-7.

**Figure 2:**
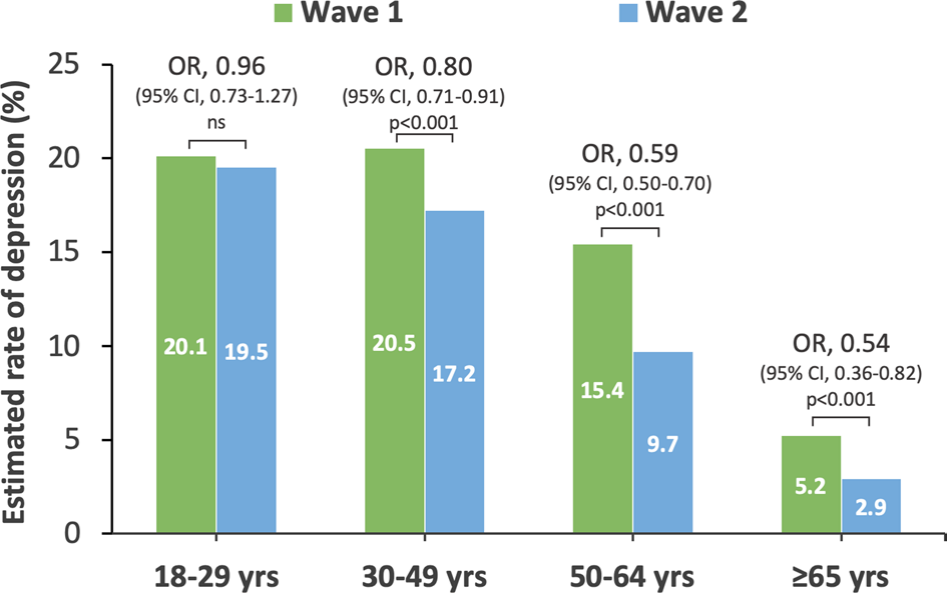
Estimated rate of depression for each age group in wave 1–2. *CI* confidence interval, *OR* odds ratio.

The highest estimated rate of depression from the first to the second wave was observed among the 18–29 years age group, and only this group demonstrated no reduction in the rate of depression (OR = 0.96, 95% CI 0.73 to 1.27, p = 0.778; Fig. 2; Table 3).

In addition, those with a history of treatment for mental illness (current, past, and both) and those with an income of < ¥2 million had particularly high estimated rates of depression (Table 4, Supplementary Table S3, Supplement A).

**Table 4:** Changes in outcome variables by income group.

N = 6112	Wave 1					Wave 2				
	< ¥2 M^a^	¥2 M to < ¥4 M	¥4 M to < ¥6 M	¥6 M to < ¥8 M	≥ ¥8 M	< ¥2 M	¥2 M to < ¥4 M	¥4 M to < ¥6 M	¥6 M to < ¥8 M	≥ ¥8 M
Depression (PHQ9 ≥ 10), n (%)	123 (28.1%)	246 (17.3%)	263 (16.8%)	153 (14.2%)	189 (11.7%)	98 (22.4%)	196 (13.8%)	184 (11.8%)	111 (10.3%)	136 (8.4%)
Suicidal ideation, n (%)	131 (29.9%)	285 (20.1%)	294 (18.8%)	196 (18.2%)	256 (15.9%)	118 (26.9%)	224 (15.8%)	250 (16.0%)	141 (13.1%)	183 (11.3%)
Psychological distress (K6 ≥ 5), n (%)	242 (55.3%)	652 (45.9%)	694 (44.4%)	474 (44.0%)	620 (38.4%)	182 (41.6%)	467 (32.9%)	490 (31.4%)	321 (29.8%)	382 (23.7%)
Somatic symptoms, mean (SD)	7.27 (6.40)	6.55 (5.42)	6.34 (5.39)	6.15 (5.33)	5.83 (5.24)	6.27 (6.75)	4.83 (5.24)	4.84 (5.25)	4.71 (5.26)	4.39 (4.99)
Loneliness, mean (SD)	26.82 (6.14)	24.08 (5.81)	23.57 (5.65)	23.33 (5.37)	22.27 (5.47)	26.99 (6.19)	24.41 (5.90)	23.75 (5.65)	23.42 (5.37)	22.47 (5.67)
Social network, mean (SD)	6.45 (5.84)	9.05 (6.00)	10.02 (5.87)	10.54 (5.94)	11.62 (6.14)	6.14 (5.64)	8.68 (5.89)	9.52 (5.83)	10.20 (5.75)	11.22 (6.21)
Anxiety (GAD7 ≥ 10), n (%)	–	–	–	–	–	66 (15.1%)	126 (8.9%)	126 (8.1%)	75 (7.0%)	97 (6.0%)
PHQ9 score, mean (SD)	6.44 (6.70)	4.73 (5.57)	4.49 (5.19)	4.17 (5.15)	3.70 (4.85)	5.62 (6.61)	3.93 (5.64)	3.66 (5.24)	3.37 (5.13)	2.89 (4.55)
K6 score, mean (SD)	7.00 (6.43)	5.36 (5.30)	5.15 (5.22)	4.90 (5.05)	4.36 (4.85)	5.46 (6.32)	3.97 (5.38)	3.69 (4.85)	3.66 (4.92)	2.96 (4.44)
GAD7 score, mean (SD)	–	–	–	–	–	3.89 (5.22)	2.88 (4.43)	2.80 (4.20)	2.63 (4.14)	2.21 (3.70)

This table shows the results excluding the 1781 participants who answered that they did not know their income.

^a^¥2 M (2 million JPY) = approximately £15,000.

*PHQ9* Patient Health Questionnaire-9, *K6* Kessler Psychological Distress Scale-6, *GAD7* Generalized Anxiety Disorder Scale-7.

### Suicidal ideation

**Figure 3:**
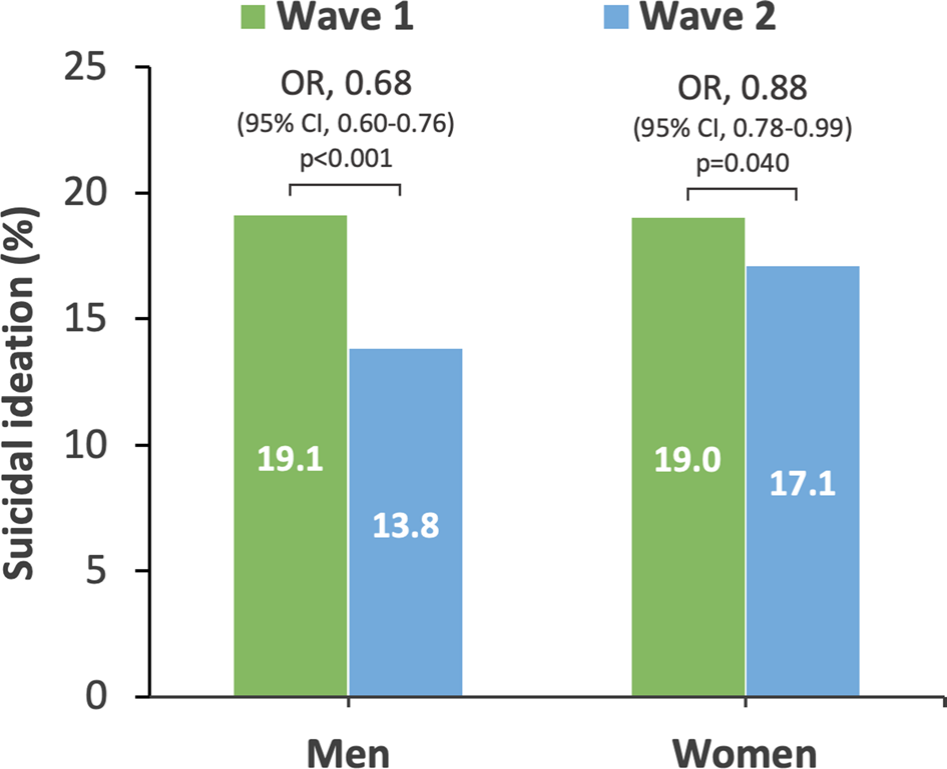
Suicidal ideation by gender in wave 1–2. *CI* confidence interval, *OR* odds ratio.

Although suicidal ideation was reduced in the second wave for both men and women (Table 3), the degree of reduction in suicidal ideation was significantly smaller among women than men (b = 0.27, 95% CI 0.14 to 0.39, p < 0.001; Fig. 3). Women also reported significantly more suicidal ideation than men in the second wave (OR = 1.29, 95% CI 1.15 to 1.46, p < 0.001), although this difference was not observed in the first wave (OR = 0.99, 95% CI 0.89 to 1.11, p = 0.907).

Only the 18–29 years age group displayed no reduction in suicidal ideation from the first to the second wave (OR = 0.99, 95% CI 0.76 to 1.29, p = 0.947; Table 3).

In addition, those with a history of treatment for mental illness and those with an income of < ¥2 million had a particularly high rate of suicidal ideation (Table 4; Supplementary Table S3, Supplement A).

### Psychological distress

All age groups displayed lower stress in the second wave (Table 3), but this reduction was significantly smaller among the 18–29 and 30–49 years age groups than the 65 years age group (18–29 years: b = 0.45, 95% CI 0.21 to 0.69, p < 0.001; 30–49 years: b = 0.32, 95% CI 0.14 to 0.49, p < 0.001). Among those aged ≥ 65 years, stress was significantly lower than in the other age groups in both waves (all p-values < 0.001).

Moreover, stress was higher among women, those with a history of treatment for mental illness, and those with an income of < ¥2 million (Tables 3 and 4; Supplementary Table S3, Supplement A).

### Somatic symptoms

Although physical symptoms decreased significantly from the first to the second wave in all age groups (Table 3), only the 18–29 years age group demonstrated a significantly smaller decrease in physical symptoms than those aged ≥ 65 years (b = 0.88, 95% CI 0.45 to 1.31, p < 0.001). Additionally, in both waves, physical symptoms were reported significantly less frequently among those aged ≥ 65 years than other age groups (all p-values < 0.001).

Further, physical symptoms decreased significantly from the first to the second wave for all income groups (Table 4), but compared with the group with an income of ≥ ¥8 million, the group with an income of < ¥2 million in the second wave displayed particularly high physical symptoms (b = 1.88, 95% CI 1.20 to 2.56, p < 0.001).

In addition, women and those with a history of treatment for mental illness showed higher physical symptoms (Table 3; Supplementary Table S3, Supplement A).

### Loneliness

Among men, loneliness did not vary between waves, (b = 0.09, 95% CI − 0.03 to 0.21, p = 0.162), whereas for women, loneliness increased significantly (b = 0.42, 95% CI 0.07 to 0.55, p < 0.001) (Table 3). Loneliness was significantly higher among men than women during both waves (wave 1: b = 0.76, 95% CI 0.51 to 1.01, p < 0.001; wave 2: b = 0.43, 95% CI 0.17 to 0.69, p = 0.001).

In addition, loneliness was higher among those aged < 65 years, those with a history of treatment for mental illness, and those with an income of < ¥2 million (Tables 3 and 4; Supplementary Table S3, Supplement A).

### Social network

From the first wave to the second wave, both men and women significantly decreased their social networks (Table 3), but the degree of decrease was significantly greater for women than for men (b = −0.43, 95% CI − 0.54 to − 0.33, p < 0.001). Additionally, in both waves, men had significantly lower social networks than women (wave 1: b = −1.36, 95% CI − 1.62 to − 1.09, p < 0.001; wave 2: b = −1.14, 95% CI − 1.40 to − 0.87, p < 0.001).

Additionally, those aged 30–64 years, those with a history of treatment for mental illness, and those with an income of < ¥2 million had lower social networks (Tables 3 and 4; Supplementary Table S3, Supplement A).

### Exhaustive interaction of factors associated with depression

**Figure 4:**
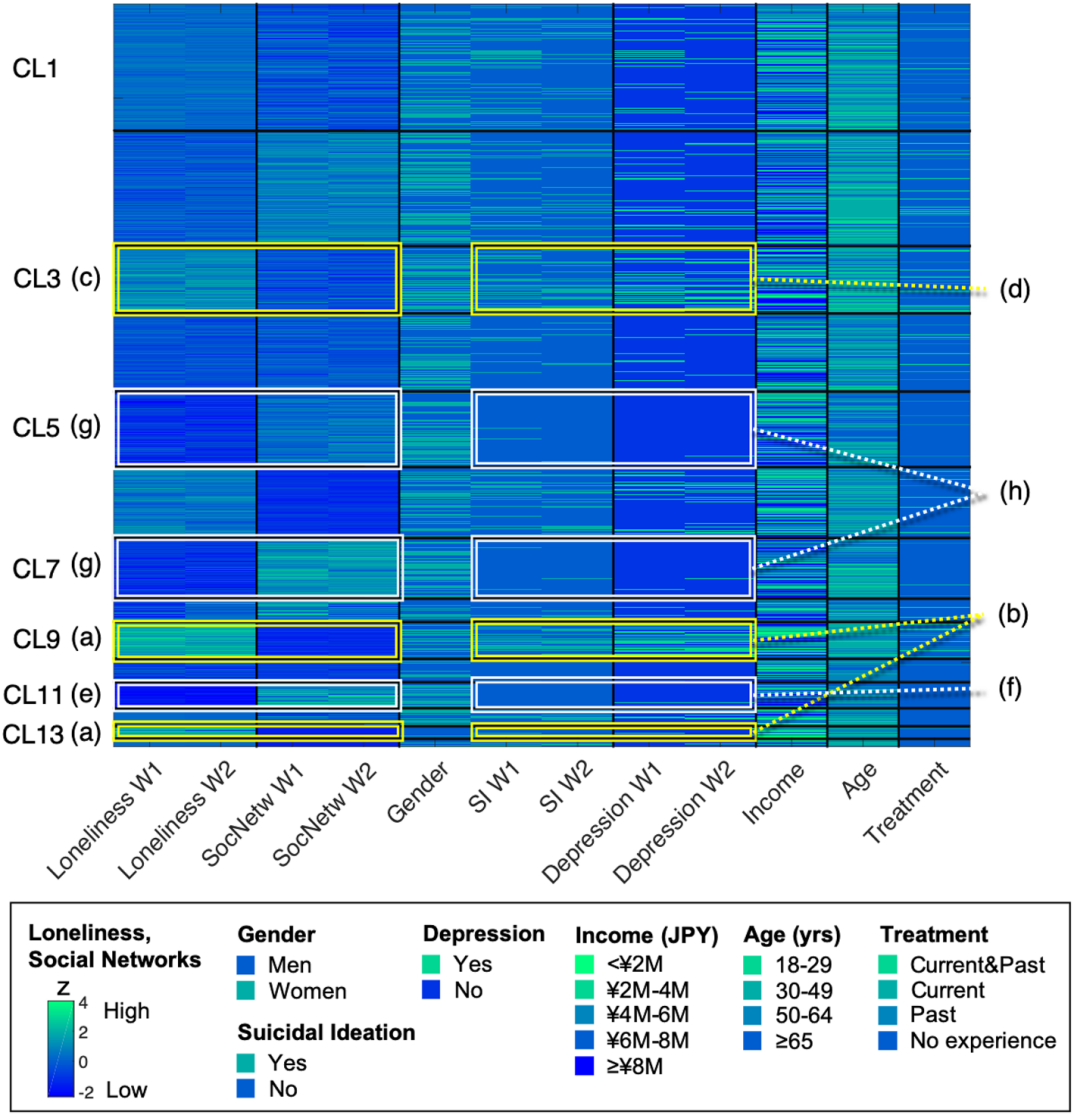
Visualization of an exhaustive interaction structure of factors associated with depression. The rows represent the data of the participants. The columns represent depression, suicidal ideation, and their associated factors. The black lines show the clusters of participants and factors, respectively. The colours in the cells indicate the magnitude of the z-values or the demographic attributes (see Box at the bottom of the figure). The appended (a), (c), (e), and (g) indicate clusters with significantly higher or lower loneliness and social networks, and (b), (d), (f), and (h) with significantly higher or lower depression and suicidal ideation. M, million; SI, suicidal ideation; SocNetw, social network; Treatment, history of treatment for mental illness; W, wave of the survey.

The final convergence results of the extracted cluster structures are shown in Fig. 4 and Supplementary Table S4.

Cluster 9 (CL9, n = 385) and CL13 (n = 136), with significantly higher loneliness and the lowest social network (Fig. 4a), had the highest estimated rates of depression in both waves (CL9: 47.8–54.8%; CL13: 35.3–42.6%), and both CLs had the highest number of people reporting suicidal ideation (CL9: 48.6–50.6%; CL13: 40.4–47.8%) (Fig. 4b). Similarly, CL3 (n = 718), the next highest in loneliness (Fig. 4c), also showed higher estimated rates of depression (28.6–37.6%) and suicidal ideation (30.4–36.8%) in both waves (Fig. 4d).

Alternatively, CL11 (n = 281), which had the lowest loneliness and also the most preserved social network (Fig. 4e), showed the lowest estimated rates of depression (0.4–0.7%) and suicidal ideation (both 0.4%) among all CLs in both waves (Fig. 4f). Similarly, CL7 (n = 637) and CL5 (n = 794), who were the next least lonely and maintained social networks (Fig. 4g), had lower estimated rates of depression (CL7: 1.9–3.3%; CL5: 2.0–2.3%) and suicidal ideation (CL7: 2.8–3.9%; CL5: 2.0–2.9%) (Fig. 4h).

## Discussion

The results of this study indicate that the overall psychological and physical symptoms decreased during the second mild lockdown compared to the first. During the first mild lockdown due to the declaration of the first-ever state of emergency in Japan, there were major economic and social disruption impacts^14^, resulting in a significant increase in depression and stress. Alternatively, difficulties in home, work, and school life were reduced in the second wave compared to the first. Further, in the second wave, both consumption activity and mobility tended to increase. Therefore, it is likely that these symptoms were reduced to some extent during the second wave, due to adaptation to various environments and changes caused by the COVID-19 pandemic.

However, at both time points of the study, social network levels indicated social isolation (Lubben Social Network Scale [LSNS-6] < 12)^15^, which was even more pronounced during the second mild lockdown. Furthermore, loneliness scores increased in the second wave compared to the first wave. These considerations indicate that repeated mild lockdowns cause cumulative changes in interpersonal interactions and that subjective loneliness is maintained and exacerbated.

A striking result when focusing on subgroups was that younger people (aged 18–29 years) displayed the highest levels of depression and suicidal ideation, and unlike other groups, these indicators did not decrease during the second wave. Additionally, the younger age group demonstrated the smallest decrease in stress and physical symptoms in the second wave of the survey. Women also indicated higher levels of depression, suicidal ideation, and increased loneliness compared to men. These results are consistent with previous reports of deteriorating mental health among young people during the pandemic^5,7,16^, indicating that younger people are more vulnerable to the negative impacts of repeated mild lockdowns. Further, considering that the estimated rate of depression among younger people in this study (22.8–24%) was significantly greater than that of the general population in Japan in the past (7.9%)^17^, the present results may indicate that young people experience difficulties with their current situation (e.g., various obstacles related to school and work, financial difficulties due to inability to work), and concerns about the future (e.g., uncertain career paths and employment status) during lockdown situations. Given these considerations, immediate support for young people is highly desirable during repeated lockdown situations. For example, support is needed from educational institutions, companies, and the government by guaranteeing educational and employment opportunities, providing mental health care, and providing information to assuage their concerns.

In addition, those with a history of treatment for mental illness and those who were socially disadvantaged (especially those with incomes < ¥2 million) had higher levels of depression, suicidal ideation, stress, physical symptoms, and loneliness, and smaller social networks at both waves. These findings are consistent with that of previous studies^18,19^ and indicate that these populations are also more susceptible to negative effects of repeated lockdowns. The COVID-19 pandemic has also been reported to cause various economic crises, resulting in lower wages, cutbacks, and general job losses in various industries^9^, making life increasingly difficult. Therefore, during repeated lockdown situations, a detailed understanding of people's difficulties with these characteristics is important, and additional social support from social workers and local governments should be considered.

Furthermore, the comprehensive extraction of interactions among variables associated with depression demonstrated that the intensity of loneliness and the size of social networks were most closely related to mental health during both mild lockdown situations. Loneliness and social networks have been considered risk factors associated with mental and physical health^20,21^ and have been identified as important factors affecting mental health during the COVID-19 pandemic^4,22^. Considering these previous studies and our results indicating that repeated mild lockdowns cumulatively reduced social networks and maintained or exacerbated loneliness, we believe that the health status of people who display reduced social networks and high loneliness requires special attention in these situations.

### Limitations

In this study, causal factors, such as which approach mitigates or exacerbates the effects of repeated mild lockdowns, have not yet been examined. Therefore, it is necessary to examine the effects of psychosocial variables, such as coping strategies and lifestyles, in the future.

Additionally, since this study used only an online survey method, the mental health of groups without online access has not yet been examined. Further, since mental health indicators were based on self-reports rather than clinical diagnoses, they may not necessarily correspond to objective assessments by mental health professionals. Therefore, it is necessary to widely use non-online methods in the future to accumulate findings that can be generalised to a larger population.

Finally, those who did not participate in the second wave of the survey had worse mental health than those who participated in both waves. Therefore, it should be noted that the present results using only those who participated in both waves may underestimate the impact of repeated lockdowns. Additionally, although the results of previous studies indicate that mental health worsened with the first mild lockdown^6,23^, we did not obtain pre-pandemic data for the participants in our study. Therefore, the exact transition in mental health needs to be judged comprehensively, taking into account the results of future studies.

## Conclusion

During the two rounds of mild lockdown in Japan, depression, suicidal ideation, stress, and physical symptoms decreased overall in the second compared to the first wave, while loneliness increased and social networks decreased. These results suggest that repeated mild lockdowns exacerbates social isolation and has a cumulative negative effect on loneliness. In addition, depression and suicidal ideation were highest among younger individuals, and as a striking result, they did not decrease in the second wave only in the younger age group. Furthermore, women, those with a history of treatment for mental illness, and those from socially disadvantaged backgrounds displayed poorer mental and physical health in both waves. These results indicate that these populations are particularly vulnerable to the negative effects of repeated mild lockdowns. Additionally, loneliness and social networks, in particular, were closely related to mental health at both time points. Given the potential for social isolation and loneliness to be exacerbated by repeated mild lockdowns, these results suggest that during a pandemic situation, people who exhibit these characteristics may require special attention. In the future, it will be essential to conduct continuous research on the health status of potentially vulnerable populations using a combination of various measures to elucidate protective and risk factors and to develop support systems tailored to individuals’ difficulties.

## Methods

### Study design, participants, and data collection

This longitudinal study was conducted online between 11 and 12 May 2020 (wave 1) and between 24 February and 1 March 2021 (wave 2). Both online surveys were conducted just before the emergency declaration was phased out, and each was set up to assess the psychological impact of a mild lockdown of approximately 1 month on participants (Fig. 1). Participants were recruited by Macromill, Inc. (Tokyo, Japan), a global marketing research company. In the first wave of the survey, approximately 80,000 people registered with Macromill, Inc. were recruited via email (the target sample was 11,000 people). A total of 11,333 people participated in the initial recruitment (the first wave), and 7893 of them participated in the follow-up recruitment (the second wave). Participants were recruited only from the seven prefectures where the emergency declaration was first applied (Tokyo, Kanagawa, Osaka, Saitama, Chiba, Hyogo, and Fukuoka prefectures) to sensitively detect the impact of mild lockdown. Since these cities have large populations and a large number of reported cases, it was assumed that they would sensitively reflect the impact of mild lockdown. The exclusion criteria for participants were: aged < 18 years, high school students, and living outside the seven prefectures.

The amount of data collected in each prefecture in the first wave was determined according to the ratio of the number of people living in each province to the total population of the seven prefectures (e.g., in the case of Tokyo, 11,428,937 (the population of Tokyo)/46,548,456 (the population of the seven prefectures) = 24.6%), Tokyo (n = 2783, 24.6%), Kanagawa (n = 1863, 16.4%), Osaka (n = 1794, 15.8%), Saitama (n = 1484, 13.1%), Chiba Prefecture (n = 1263, 11.1%), Hyogo (n = 1119, 9.9%), and Fukuoka (n = 1027, 9.1%). The data from the first wave of the survey in this study are part of the dataset used in previous publications^6,11^, and details of this data can be found in these papers.

A link to an online platform to participate in the survey was distributed to those who wished to participate in the study, and the survey was conducted online. All participants completed the anonymous survey voluntarily and provided informed consent online. The online survey form was designed so that all items were required to be answered before proceeding to the next step; thus, there were no missing data in this study. The survey procedures were clearly explained, and participants could interrupt or terminate the survey at any time without providing an explanation. This study was approved by the Research Ethics Committee of the Graduate School of Social and Industrial Science and Technology, Tokushima University (approval number: 212) and has been performed in accordance with the ethical standards laid down in the 1964 Declaration of Helsinki and its later amendments.

### Measures

Depressive symptoms were assessed using the Japanese version of the PHQ-9 ^24^, which consists of nine items; a score of ≥ 10 was considered to indicate a high likelihood of depression^24^. To show the trend of the percentage of people with severe symptoms and to allow comparison with previous studies^3,5^, we used this as a cut-off point in this study.

Suicidal ideation was assessed using item 9 of the PHQ-9 and coded as a binary variable, with a response of “not at all” as “no suicidal ideation” and all other responses as “suicidal ideation”.

Psychological distress was measured using the Japanese version of the K6^25^, which consists of six items. A threshold of five points, commonly used for screening mild to moderate mood/anxiety disorders, was adopted as the cut-off point for psychological distress^26^.

Somatic symptoms were measured using the Japanese version of the Somatic Symptom Scale-8^27^, which consists of eight items.

Loneliness was measured using the 10-item Japanese version of the UCLA Loneliness Scale Version 3^28^.

Social networks and the presence of social support were measured using the Japanese version of the abbreviated LSNS-6^15^, which consists of six items. LSNS-6 scores < 12 indicate social isolation, and higher scores indicate a larger social network.

The Japanese version of the GAD-7^29^, consisting of seven items, was used to assess symptoms of generalised anxiety disorder. Scores > 10 are considered to indicate a moderate level of anxiety^30^ and were used as a cut-off point in this study. Since the GAD-7 was used only in the second wave, it was used to describe the characteristics of participants in the second wave.

These self-report measures are more extensively described in Supplement B in the Supplementary Materials.

To account for differences in living conditions between waves 1 and 2, we asked respondents to rate the frequency of experiencing “household financial deterioration”, “living difficulties”, and “academic or occupational difficulties” from the start of mild lockdown to the time of the survey on a scale of 1 (not at all) to 7 (extremely), based on Yamamoto et al.^6^. Similarly, to examine differences in social self-restraint, consumption activity (nominal consumption activity index), and going-out behavior (public transportation congestion) in waves 1 and 2 were calculated based on basic data from the Bank of Japan^31^ and Google Community Mobility Reports^32^, respectively.

Further, to determine the influence of mild lockdowns on groups that have been previously identified as vulnerable to the effects of lockdown^6,12,13^, demographic attributes, such as age, gender, and income, were collected, as well as whether or not individuals were currently being treated for mental illness and whether or not they had a history of treatment for a mental illness.

### Statistical analysis

Analyses were performed on a dataset of 7893 participants from both waves of the survey. First, comparisons were made using t-tests and chi-square tests for the data in the first wave for those who participated in both surveys (N = 7893) and those who only participated in the first wave survey (N = 3440).

Next, life and academic/occupational difficulties in the first and second waves were compared using t-tests.

For each outcome variable, a generalised estimating model (GEE) was constructed to test for changes in the variable between waves for the whole sample. This approach is applicable to binary variables and variables other than normally distributed ones and has excellent analytical precision, even for longitudinal data with high within-subject correlation^33,34^. In the GEE approach, binomial logit modelling was used for the binary outcome variables (PHQ-9 cut-off score, suicidal ideation, and K6 cut-off score), and linear Gaussian identity modelling was used for the continuous outcome variables (physical symptoms, loneliness, and social network).

Additionally, GEE models were used to examine differences in changes in outcome variables in subgroups focused on gender (men, women), age (18–29, 30–49, 50–64, ≥ 65 years), income (< ¥2 million (approximately £15,000), ¥2–4 million, ¥4–6 million, ¥6–8 million, ≥ ¥8 million), and treatment for mental illness (currently in treatment, past treatment, past treatment and still in treatment, no treatment). For analysis of the whole sample, binary outcome variables were assessed using a binomial logit GEE model, and continuous outcome variables were assessed using a linear Gaussian identity GEE model. Based on Ballinger^33^, the Wald test was used to examine the significance of the model. Significant interactions between each subgroup and wave were mainly reported. When analysing the subgroups of income groups, we excluded 1781 participants who answered that they did not know.

To visualise the interaction structure of the variables most closely associated with depression, we used nonparametric Bayesian co-clustering^35^ for gender, age, income, history of treatment for mental illness, depression, suicidal ideation, loneliness, and social networks. Since this method is capable of exhaustively extracting various clusters represented by multidimensional factors^6,35^, it is expected to find the most influential factors for depression. A total of 15,000 iterations based on the Bayesian optimization principle were performed, and the marginal log-likelihood (Bayesian factor) was calculated as an objective criterion for good classification. After confirming that the marginal log-likelihood was completely stable, the model with the highest marginal log-likelihood was finally adopted. This procedure allowed us to select a classification result with high objectivity and reproducibility.

The significance level for all tests was set at α = 0.05, two-tailed. The analysis was performed using Matlab R2017a (Mathworks Inc.) and SPSS (version 22.0; SPSS Japan Inc., Tokyo, Japan).

### Ethical standard

This study was approved by the Research Ethics Committee of the Graduate School of Social and Industrial Science and Technology, Tokushima University (approval number: 212) and has been performed in accordance with the ethical standards laid down in the 1964 Declaration of Helsinki and its later amendments.

## Supplementary Information

Supplementary Information.
