# Supplementary material for: Mental health and social isolation under repeated mild lockdowns in Japan
Source: Sci Rep. 2022 May 19;12:8452. doi: 10.1038/s41598-022-12420-0 (PMC9118820; doi:10.1038/s41598-022-12420-0)
Supplement: Supplementary file 1 — Supplementary Information. [file 41598_2022_12420_MOESM1_ESM.pdf]

Mental health and social isolation under repeated mild lockdowns in Japan

Supplementary Materials

Contents

Supplementary Table S1. Comparison between those who participated in all surveys and those who participated in wave 1 survey only 2

Supplementary Table S2. Changes in difficulties in household economy, living, work or schoolwork 3

Supplementary Table S3. Changes in outcome variables by history of treatment for mental illness 4

Supplementary Table S4. Comprehensive interaction structure of variables associated with depression 5

Supplementary Figure S1. Trends in consumption activity in Japan from February 2020 to April 2021 7

Supplementary Figure S2. Trends in public transportation congestion in Japan from February 2020 to April 2021 8

Supplement A. Full Details of Results 9

Supplement B. Full Details of Measures 12

Supplementary References 14

Supplementary Table S1. Comparison between those who participated in all surveys and those who participated in wave 1 survey only

	Participated in all surveys (n=7,893)	Participated in wave 1 only (n=3,440)	χ2 or t	df	p	d or V
Age, mean (SD)	49.56 (13.71)	38.66 (13.55)	39.3	6,621	< 0.001	0.80
Women, n (%)	3,692 (46.8%)	2,250 (65.4%)	333.4	1	< 0.001	0.17
Married, n (%)	5,174 (65.6%)	1,869 (54.3%)	128.2	1	< 0.001	0.11
Depression (PHQ9 ≥ 10), n (%)	1,286 (16.3%)	748 (21.7%)	48.3	1	< 0.001	0.07
Suicidal ideation, n (%)	1,503 (19.0%)	754 (21.9%)	12.4	1	< 0.001	0.03
Psychological Distress (K6 ≥ 5), n (%)	3,595 (45.5%)	1,854 (53.9%)	66.9	1	< 0.001	0.08
Somatic Symptoms, mean (SD)	6.39 (5.50)	7.37 (5.79)	8.49	6,261	< 0.001	0.18
Loneliness, mean (SD)	23.59 (5.74)	23.17 (5.59)	3.60	6,717	< 0.001	0.07
Social Network, mean (SD)	10.14 (6.13)	11.53 (6.15)	11.1	6,528	< 0.001	0.23

PHQ9, Patient Health Questionnaire-9; K6, Kessler Psychological Distress Scale-6.

Supplementary Table S2. Changes in difficulties in household economy, living, work or schoolwork

N=7,893	Wave 1	Wave 2	t	df	p	d
Deterioration of household economy, mean (SD)	3.72 (1.77)	3.45 (1.74)	12.8	7,892	< 0.001	0.15
Difficulties owing to the lack of daily necessities, mean (SD)	3.57 (1.83)	2.60 (1.59)	41.7	7,892	< 0.001	0.57
Difficulties in work or schoolwork, mean (SD)	3.62 (1.98)	2.85 (1.75)	33.5	7,892	< 0.001	0.41

Supplementary Table S3. Changes in outcome variables by history of treatment for mental illness

	Wave 1				Wave 2			
N=7,893	Current (n=118)	Past (n=562)	Current & Past (n=313)	No (n=6,900)	Current (n=118)	Past (n=562)	Current & Past (n=313)	No (n=6,900)
Depression (PHQ9 ≥ 10), n (%)	66 (55.9%)	156 (27.8%)	170 (54.3%)	894 (13.0%)	46 (39.0%)	128 (22.8%)	152 (48.6%)	663 (9.6%)
Suicidal ideation, n (%)	59 (50.0%)	176 (31.3%)	159 (50.8%)	1,109 (16.1%)	51 (43.2%)	149 (26.5%)	149 (47.6%)	863 (12.5%)
Psychological Distress (K6 ≥ 5), n (%)	98 (83.1%)	374 (66.5%)	250 (79.9%)	2,873 (41.6%)	83 (70.3%)	283 (50.4%)	219 (70.0%)	1,922 (27.9%)
Somatic Symptoms, mean (SD)	11.47 (5.63)	8.98 (5.56)	11.98 (6.91)	5.83 (5.17)	9.54 (6.47)	7.09 (6.00)	10.83 (7.57)	4.37 (4.94)
Loneliness, mean (SD)	26.28 (5.26)	25.29 (5.90)	27.34 (6.37)	23.23 (5.61)	26.69 (5.70)	25.63 (6.00)	27.52 (6.57)	23.46 (5.71)
Social Network, mean (SD)	7.78 (5.48)	9.04 (5.50)	7.68 (5.34)	10.38 (6.18)	7.61 (5.83)	8.58 (5.27)	7.46 (5.53)	9.94 (6.11)
Anxiety (GAD7 ≥ 10), n (%)	-	-	-	-	39 (33.1%)	89 (15.8%)	113 (36.1%)	426 (6.2%)
PHQ9 score, mean (SD)	11.03 (6.56)	7.11 (5.80)	11.04 (7.03)	3.92 (4.90)	8.69 (7.08)	6.14 (6.00)	10.41 (7.66)	3.20 (4.86)
K6 score, mean (SD)	11.08 (5.96)	7.84 (5.78)	10.71 (6.71)	4.72 (4.94)	8.99 (6.50)	5.99 (5.73)	9.29 (7.14)	3.34 (4.67)
GAD7 score, mean (SD)	-	-	-	-	6.68 (5.35)	4.81 (4.93)	7.91 (6.40)	2.38 (3.83)

PHQ9, Patient Health Questionnaire-9; K6, Kessler Psychological Distress Scale-6; GAD7, Generalized Anxiety Disorder Scale-7.

Supplementary Table S4. Comprehensive interaction structure of variables associated with depression

N= 7,893	CL 1 (n=1,344)	CL2 (n=1,225)	CL3 (n=718)	CL4 (n=835)	CL5 (n=794)	CL6 (n=768)	CL7 (n=637)	CL8 (n=247)	CL9 (n=385)	CL10 (n=252)	CL11 (n=281)	CL12 (n=180)	CL13 (n=136)	CL14 (n=91)
Depression (PHQ9 ≥ 10) wave 1, n (%)	239 (17.8%)	180 (14.7%)	270 (37.6%)	49 (5.9%)	18 (2.3%)	166 (21.6%)	12 (1.9%)	39 (15.8%)	211 (54.8%)	7 (2.8%)	1 (0.4%)	26 (14.4%)	58 (42.6%)	10 (11.0%)
Depression (PHQ9 ≥ 10) wave 2, n (%)	171 (12.7%)	121 (9.9%)	205 (28.6%)	35 (4.2%)	16 (2.0%)	123 (16.0%)	21 (3.3%)	25 (10.1%)	184 (47.8%)	6 (2.4%)	2 (0.7%)	18 (10.0%)	48 (35.3%)	14 (15.4%)
Suicidal ideation wave 1, n (%)	344 (25.6%)	208 (17.0%)	264 (36.8%)	66 (7.9%)	23 (2.9%)	200 (26.0%)	18 (2.8%)	48 (19.4%)	195 (50.6%)	8 (3.2%)	1 (0.4%)	44 (24.4%)	65 (47.8%)	19 (20.9%)
Suicidal ideation wave 2, n (%)	244 (18.2%)	153 (12.5%)	218 (30.4%)	47 (5.6%)	16 (2.0%)	164 (21.4%)	25 (3.9%)	30 (12.1%)	187 (48.6%)	12 (4.8%)	1 (0.4%)	39 (21.7%)	55 (40.4%)	21 (23.1%)
Loneliness wave 1, z value	0.32	-0.11	1.07	-0.33	-1.12	0.66	-1.06	-0.23	1.90	-0.63	-1.95	0.25	1.72	0.31
Loneliness wave 2, z value	0.31	-0.09	1.08	-0.34	-1.13	0.63	-1.03	-0.18	1.92	-0.65	-1.96	0.20	1.64	0.29
Social Network wave 1, z value	-0.29	0.77	-0.33	-0.21	0.35	-1.16	1.42	0.91	-1.19	-0.75	1.45	-0.24	-1.65	-1.65
Social Network wave 2, z value	-0.32	0.75	-0.38	-0.20	0.39	-1.16	1.41	1.08	-1.19	-0.79	1.50	-0.19	-1.60	-1.60
**Gender**, n (%)														
Women	593 (44.1%)	642 (52.4%)	384 (53.5%)	385 (46.1%)	411 (51.8%)	268 (34.9%)	351 (55.1%)	92 (37.2%)	181 (47.0%)	103 (40.9%)	161 (57.3%)	63 (35.0%)	38 (27.9%)	20 (22.0%)
**Age**, n (%)														
18-29 yrs	87 (6.5%)	147 (12.0%)	88 (12.3%)	36 (4.3%)	61 (7.7%)	28 (3.6%)	57 (8.9%)	43 (17.4%)	31 (8.1%)	11 (4.4%)	17 (6.0%)	21 (11.7%)	6 (4.4%)	4 (4.4%)
30-49 yrs	627 (46.7%)	605 (49.4%)	413 (57.5%)	304 (36.4%)	236 (29.7%)	276 (35.9%)	236 (37.0%)	59 (23.9%)	216 (56.1%)	68 (27.0%)	94 (33.5%)	73 (40.6%)	62 (45.6%)	33 (36.3%)
50-64 yrs	468 (34.8%)	311 (25.4%)	167 (23.3%)	345 (41.3%)	288 (36.3%)	373 (48.6%)	181 (28.4%)	111 (44.9%)	118 (30.6%)	100 (39.7%)	76 (27.0%)	65 (36.1%)	55 (40.4%)	43 (47.3%)
≥65 yrs	162 (12.1%)	162 (13.2%)	50 (7.0%)	150 (18.0%)	209 (26.3%)	91 (11.8%)	163 (25.6%)	34 (13.8%)	20 (5.2%)	73 (29.0%)	94 (33.5%)	21 (11.7%)	13 (9.6%)	11 (12.1%)
**Treatment**, n (%)														
Current	37 (2.8%)	15 (1.2%)	13 (1.8%)	3 (0.4%)	4 (0.5%)	19 (2.5%)	4 (0.6%)	2 (0.8%)	9 (2.3%)	5 (2.0%)	0 (0.0%)	1 (0.6%)	4 (2.9%)	2 (2.2%)
Past	135 (10.0%)	73 (6.0%)	76 (10.6%)	49 (5.9%)	47 (5.9%)	52 (6.8%)	25 (3.9%)	10 (4.0%)	48 (12.5%)	15 (6.0%)	9 (3.2%)	8 (4.4%)	10 (7.4%)	5 (5.5%)
Current+Past	18 (1.3%)	36 (2.9%)	64 (8.9%)	33 (4.0%)	20 (2.5%)	43 (5.6%)	3 (0.5%)	14 (5.7%)	61 (15.8%)	1 (0.4%)	4 (1.4%)	4 (2.2%)	11 (8.1%)	1 (1.1%)
No experience	1154 (85.9%)	1101 (89.9%)	565 (78.7%)	750 (89.8%)	723 (91.1%)	654 (85.2%)	605 (95.0%)	221 (89.5%)	267 (69.4%)	231 (91.7%)	268 (95.4%)	167 (92.8%)	111 (81.6%)	83 (91.2%)
**Income**[a], n (%)														

<¥2M[b]	54 (5.1%)	40 (4.4%)	40 (7.5%)	14 (2.2%)	22 (3.4%)	73 (12.1%)	7 (1.5%)	10 (5.1%)	77 (27.2%)	33 (15.6%)	5 (2.2%)	12 (8.8%)	31 (27.9%)	20 (27.4%)
¥2M to <¥4M	246 (23.1%)	151 (16.6%)	134 (25.0%)	166 (26.1%)	150 (23.4%)	192 (31.7%)	79 (16.5%)	42 (21.2%)	82 (29.0%)	61 (28.8%)	40 (17.6%)	22 (16.1%)	36 (32.4%)	20 (27.4%)
¥4M to <¥6M	315 (29.5%)	227 (25.0%)	162 (30.2%)	166 (26.1%)	151 (23.5%)	139 (23.0%)	121 (25.2%)	47 (23.7%)	57 (20.1%)	49 (23.1%)	51 (22.5%)	42 (30.7%)	22 (19.8%)	13 (17.8%)
¥6M to <¥8M	186 (17.4%)	217 (23.9%)	102 (19.0%)	140 (22.0%)	105 (16.4%)	96 (15.9%)	93 (19.4%)	30 (15.2%)	30 (10.6%)	9 (4.2%)	34 (15.0%)	19 (13.9%)	8 (7.2%)	9 (12.3%)
≥8M	265 (24.9%)	272 (30.0%)	98 (18.3%)	149 (23.5%)	214 (33.3%)	105 (17.4%)	180 (37.5%)	69 (34.8%)	37 (13.1%)	60 (28.3%)	97 (42.7%)	42 (30.7%)	14 (12.6%)	11 (15.1%)

[a] Rows of Income category show the results excluding the 1,781 participants who answered that they did not know their income.

[b] ¥2M (2 million JPY) = approximately £15,000.

CL, participants cluster; PHQ9, Patient Health Questionnaire-9.

Supplementary Figure S1. Trends in consumption activity in Japan from February 2020 to April 2021

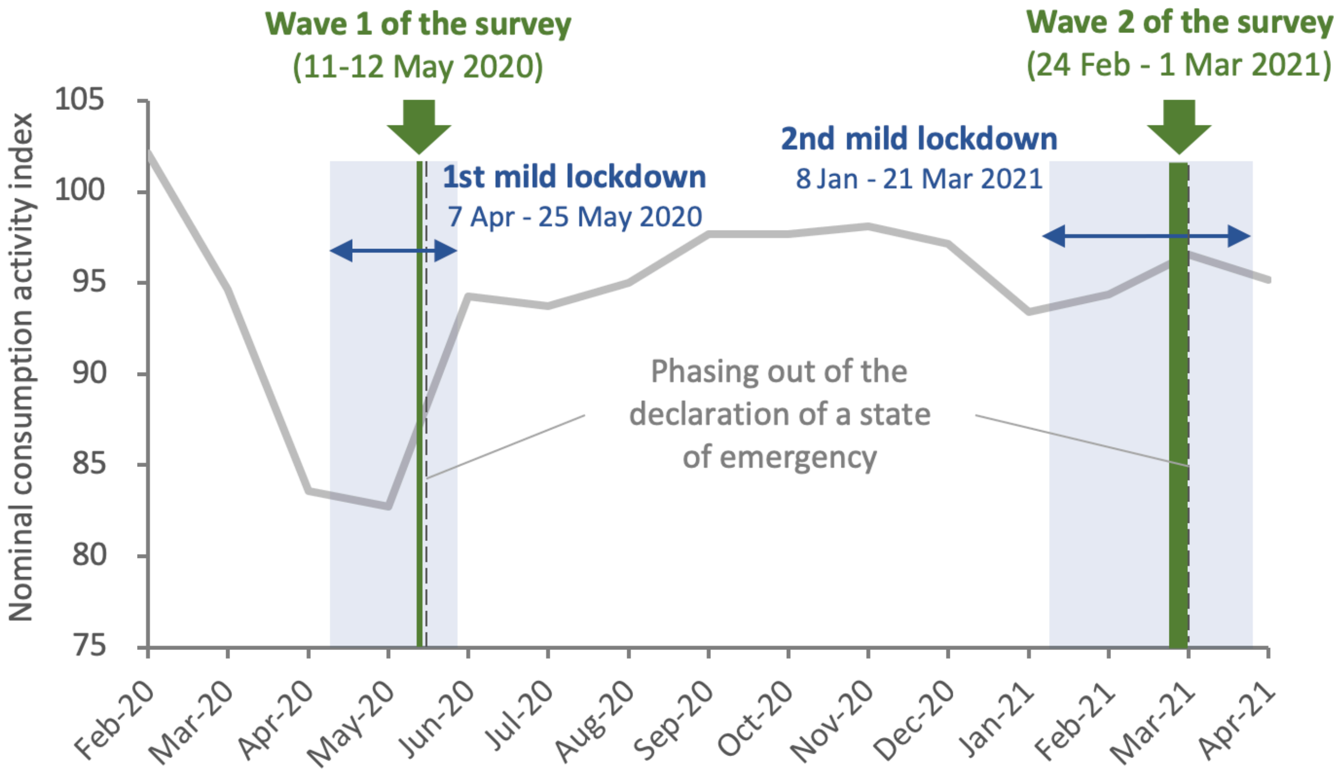

For the baseline of the nominal consumption activity index, we used 2015 data based on the Bank of Japan's calculation methodology.

Supplementary Figure S2. Trends in public transportation congestion in Japan from February 2020 to April 2021

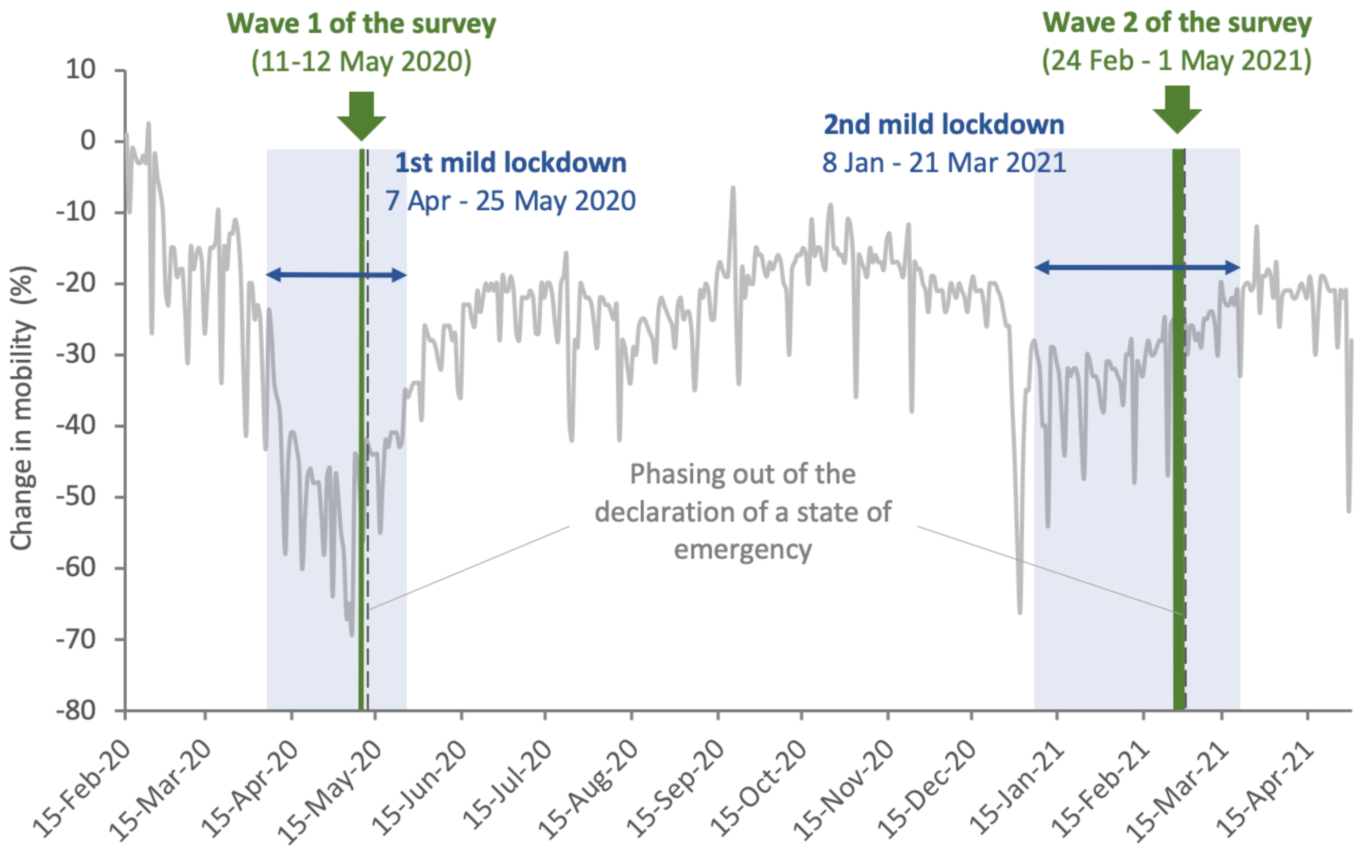

The vertical axis indicates the number of visits and time spent on public transportation. As a baseline, the median values for each day of the week from January 3 to February 6, 2020, were used.

Supplement A. Full Details of Results

Mental health under repeated mild lockdowns with a focus on subgroups

The first- and second-wave data for all subgroups are included in the Tables 3-4 and Supplementary Table S3.

Estimated rate of depression

There was a significant interaction between gender and wave, and between each age group and wave, for the estimated rate of depression as assessed by the PHQ-9 (Gender: Wald $\chi2(1) = 7.49$, $p = .006$; Age: $\chi2(3) = 24.70$, $p < .001$). Both men and women displayed a reduction in depression rate in the second wave (Table 3). However, this reduction was significantly smaller among women than men (b = 0.18, 95% CI = 0.52 to 0.32, $p < 0.001$). Women also had a significantly higher estimated rate of depression in both the first and second waves than men (wave 1: OR = 1.30, 95% CI = 1.15 to 1.46, $p < 0.001$; wave 2: OR = 1.56, 95% CI = 1.36 to 1.78, $p < 0.001$). The highest estimated rate of depression from the first to the second wave was observed among 18-29-year-olds, and only this group demonstrated no reduction in the rate of depression (OR = 0.96, 95% CI = 0.73 to 1.27, p = 0.778; Figure 2, Table 3). Additionally, among those aged 65 years and older, the rate of depression was significantly lower in both waves than the other age groups.

The estimated rate of depression was significantly higher among participants with a history of treatment for mental illness (current, past, current, and past) compared to those without (ORs = 2.58 to 8.53; all ps < 0.001; Supplementary Table S3). The estimated depression rate was significantly higher among participants with an income less than ¥8 million (< ¥2 million, ¥2-4 million, and ¥4-6 million) than those with an income ≥ ¥8 million (ORs = 1.53 to 2.94, all ps < 0.001; Table 4), and was especially highest among those with an income < ¥2 million.

Suicidal Ideation

There was a significant interaction between gender and wave, and between each age group and wave, for suicidal ideation (Gender: Wald $\chi2(1) = 17.87$, $p < 0.001$; Age: $\chi2(3) = 26.27$, $p < 0.001$). Although suicidal ideation was reduced in the second wave for both men and women (Table 3), the degree of reduction in suicidal ideation was significantly smaller among women than men (b = 0.27, 95% CI = 0.14 to 0.39, $p < 0.001$; Figure 3). Women also reported significantly more suicidal ideation than men in the second wave (OR = 1.29, 95% CI = 1.15 to 1.46, $p < 0.001$), although this difference was not observed in the first wave (OR = 0.99, 95% CI = 0.89 to 1.11, p = 0.907). Only 18-29-year olds displayed no reduction in suicidal ideation from the first to the second wave (OR = 0.99, 95% CI = 0.76 to 1.29, p = 0.947; Table 3). Additionally, suicidal ideation was significantly lower in both waves among those aged ≥65 than the other age groups (all ps < 0.001).

There were significantly more suicidal ideations among those with a history of treatment compared to those with no history of treatment (ORs=2.38-5.39, all ps < 0.001; Supplementary Table S3), and significantly more among those with an income of <¥8 million compared to those with an income of ≥¥8 million (ORs=1.23 to 2.23, all ps < 0.05; Table 4).

Psychological Distress

There was a significant interaction between age group and wave for the prevalence of stressed ratings according to the K6 (Wald $\chi2(3) = 19.98$, $p < .001$). All age groups displayed lower stress in the second wave (Table 3), but this reduction was significantly smaller among 18-29 and 30-49-year-olds than the 65-year-olds (18-29 years: b = 0.45, 95% CI = 0.21 to 0.69, $p < 0.001$; 30-49 years: b = 0.32, 95% CI = 0.14 to 0.49, $p < 0.001$). Among those aged ≥65, stress was significantly lower than in the other age groups in both waves (all ps < 0.001).

Stress was significantly higher among women (OR = 1.55, 95% CI = 1.42-1.70, $p < 0.001$), those with a history of treatment for mental illness (ORs = 2.79 to 6.87, all ps < 0.001), and those with an income of < ¥8 million (ORs = 1.26 to 1.98, all ps < 0.005) compared to men, those without a history of treatment for mental illness, and those with an income of ≥¥8 million, respectively (Tables 3-4, Supplementary Table S3).

Somatic Symptoms

There was a significant interaction in terms of age and income group (Age: Wald $\chi2(3) = 33.82$, $p < 0.001$; Income: $\chi2(4) = 9.70$, $p = 0.046$). Although physical symptoms decreased significantly from the first to the second wave in all age groups (Table 3), only 18-29-year-olds demonstrated a significantly smaller decrease in physical symptoms than those aged ≥65 years (b = 0.88, 95% CI = 0.45 to 1.31, $p < 0.001$). Additionally, in both waves, physical symptoms were reported significantly less frequently among those aged ≥65 years than other age groups (all ps < 0.001). Next, physical symptoms decreased significantly from the first to the second wave for all income groups (Table 4), but compared with the group with an income of ≥¥8 million, the group with an income of <¥2 million in the second wave displayed particularly high physical symptoms (b = 1.88, 95% CI = 1.20 to 2.56, $p < 0.001$), and the groups with an income of 2-4 million and 4-6 million displayed a similarly high trend (¥2-4 million: b = 0.43, 95% CI = 0.07 to 0.80, $p = 0.020$; ¥4-6 million: b = 0. 44, 95% CI = 0.09 to 0.80, $p = 0.015$).

Physical symptoms were higher among women (b = 0.87, 95% CI = 0.63 to 1.11, $p < 0.001$) and those with a history of treatment for mental illness (bs = 3.14 to 6.15, all ps < 0.001) than men and those without a history of treatment for mental illness. (Table 3, Supplementary Table S3).

Loneliness

A significant interaction was observed for gender and wave (Wald $\chi2(1) = 13.70$, $p < 0.001$). Among men loneliness did not vary from between waves, (b = 0.09, 95% CI = -0.03 to 0.21, $p = 0.162$), whereas for women loneliness increased significantly (b = 0.42, 95% CI = 0.07 to 0.55, $p < 0.001$)

(Table 3). Loneliness was significantly higher among men than women during both waves (wave1: $b = 0.76$, 95% CI = 0.51 to 1.01, $p < 0.001$; wave2: $b = 0.43$, 95% CI = 0.17 to 0.69, $p = 0.001$).

Those <65 years of age (bs = 2.40 to 4.55, all ps < 0.001), those with a history of treatment for mental illness (bs = 2.06 to 4.11, all ps < 0.001), and those with an income of < ¥8 million (bs = 1.06 to 4.55, all ps < 0.001) had significantly higher loneliness than those over 65 years of age, those without a history of treatment for mental illness, and those with an income of ≥¥8 million, respectively (Tables 3-4, Supplementary Table S3).

Social Network

A significant interaction was observed for gender and wave regarding social networks (Wald $\chi 2(1) = 4.28$, $p = 0.039$). From the first wave to the second wave, both men and women significantly decreased their social networks (Table 3), but the degree of decrease was significantly greater for women than for men ($b = -0.43$, 95% CI = -0.54- -0.33, $p < 0.001$). Additionally, in both waves, men had significantly lower social networks than women (wave 1: $b = -1.36$, 95% CI = -1.62 to -1.09, $p < 0.001$; wave 2: $b = -1.14$, 95% CI = -1.40 to -0.87, $p < 0.001$).

The social network was smaller for those aged 30-64 years (bs = -1.74 to -1.37, all ps < 0.001), those with a history of treatment for mental illness (bs = -1.34 to -2.69, all ps < 0.001), and those with an income of <¥8 million (bs = -1.08 to -5.17, all ps < 0.001) than for those aged ≥65, those without a history of treatment for mental illness, and those with an income of ≥¥8 million (Tables 3-4, Supplementary Table S3).

Supplement B. Full Details of Measures

Depression and Suicidal Ideation

Depression was measured by the Japanese version of the Patient Health Questionnaire-9 (PHQ-9)(Kumiko Muramatsu et al., 2018). The PHQ-9 consists of nine questions, and participants reported depressive symptoms during the past four weeks assessed by a score of 0 (not at all) to 3 (nearly every day) (Kroenke, Spitzer, & Williams, 2001). Scores range from 0 to 27 points. We defined a score of ≥10, previously recommended (Kumiko Muramatsu et al., 2018), as a cut-point, meaning that a person is more likely to have major depression. The PHQ-9 is widely used internationally as a screening scale for depression (Siu et al., 2016) with high reliability and validity (Kumiko Muramatsu et al., 2018).

For identifying suicidal ideation (SI), the item 9 of the PHQ-9 was used. The item states, "Over the last 2 weeks, how often have you been bothered by the following problem: thoughts that you would be better off dead, or of hurting yourself in some way?" Answer choices are “not at all”, “several days”, “more than half the days”, or “nearly every day”. SI was coded as a binary variable and considered present for any response other than “not at all”.

Psychological Distress and Somatic Symptom

Psychological distress was measured by the Japanese version of the K6 (Furukawa, Kessler, Slade, & Andrews, 2003), a six-item screening scale of nonspecific psychological stress in the past 30 days. Each question was rated on a scale of 0 (none of the time) to 4 (all of the time): total scores range from 0 to 24. Given its brevity and high accuracy, the K6 is an ideal scale for screening for mental disorders in population-based health surveys (Furukawa et al., 2003; Kessler, Barker, et al., 2003; Veldhuizen, Cairney, Kurdyak, & Streiner, 2007). We adopted a threshold of five points commonly used to screen for mild-to-moderate mood/anxiety disorders (MMPD) (Prochaska, Sung, Max, Shi, & Ong, 2012). This threshold is the optimal lower threshold cut-point for screening for moderate psychological distress (Prochaska et al., 2012). MMPD was assessed given the risk of progression to more severe disability as well as current distress and disability (Kessler, Merikangas, et al., 2003). Based on 4 years of published data concerning K6 from the Ministry of Health, Labor and Welfare (Ministry of Health Labour and Welfare, 2020), we defined K6 ≥ 5 as ‘psychological distress’.

Somatic symptom burden was assessed by the Japanese version of the Somatic Symptom Scale-8 (SSS-8) (Matsudaira et al., 2016), which consists of 8 items. It consists of questions about common somatic symptoms such as stomach or bowel problems and Headaches in the past week, and requires a response scale of 0 (not at all) to 4 (very much). Scores range from 0 to 32 points. The SSS-8 is highly reliable and valid for assessing somatic symptom burden (Gierk et al., 2014).

Loneliness and Social Networks

We measured loneliness and social networks using the 10-item Japanese version of the UCLA loneliness scale version 3 (UCLA-LS3) (Arimoto & Tadaka, 2019) and the Japanese version of the abbreviated Lubben Social Network Scale (LSNS-6) (Kurimoto et al., 2011), respectively.

The UCLA-LS3 consists of 10 items, each rated from 1 (never) to 4 (always) (Russell, 1996). The scores range from 10 to 40, with higher scores indicating higher levels of loneliness. The UCLA-LS3 is highly reliable and valid (Arimoto & Tadaka, 2019) and is internationally used for measuring loneliness (Durak & Senol-Durak, 2010; Shevlin, Murphy, & Murphy, 2015; Zarei, Memari, Moshayedi, & Shayestehfar, 2016).

The LSNS-6 consists of three items related to family networks and three items related to friendship networks. The number of people in the network is calculated using a six-point scale (0 = none to 5 = nine or more) for each item (Lubben, 1988). Scores range from 0 to 30 points, with higher scores indicating a larger social network and <12 points indicating social isolation. The LSNS-6 is highly reliable and valid (Kurimoto et al., 2011) and has been used in many countries (Ceria et al., 2001; Martire, Schulz, Mittelmark, & Newsom, 1999; Okwumabua, Baker, Wong, & Pilgram, 1997).

Anxiety

Anxiety was measured by the Japanese version of the Generalized Anxiety Disorder Scale-7 (GAD-7), a 7-item screening tool. The GAD-7 assesses the frequency with which the seven symptoms of anxiety occurred over the last two weeks (K Muramatsu et al., 2010) by using a scale from 0 (not at all) to 3 (nearly every day): total scores range from 0 to 21. Scores above 10 are considered to indicate a moderate level of anxiety (Spitzer, Kroenke, Williams, & Löwe, 2006) and were used as a cutoff point in this study. The GAD-7 has high reliability and validity for assessing anxiety symptoms (Ruiz et al., 2011).

Supplementary References

Arimoto, A., & Tadaka, E. (2019). Reliability and validity of Japanese versions of the UCLA loneliness scale version 3 for use among mothers with infants and toddlers: A cross-sectional study. *BMC Women's Health*, *19*(1), 105. https://doi.org/10.1186/s12905-019-0792-4

Ceria, C. D., Masaki, K. H., Rodriguez, B. L., Chen, R., Yano, K., & David Curb, J. (2001). The relationship of psychosocial factors to total mortality among older Japanese-American men: The Honolulu Heart Program. *Journal of the American Geriatrics Society*, *49*(6), 725–731. https://doi.org/10.1046/j.1532-5415.2001.49148.x

Durak, M., & Senol-Durak, E. (2010). Psychometric qualities of the ucla loneliness scale-version 3 as applied in a turkish culture. *Educational Gerontology*, *36*, 988–1007. https://doi.org/10.1080/03601271003756628

Furukawa, T. A., Kessler, R. C., Slade, T., & Andrews, G. (2003). The performance of the K6 and K10 screening scales for psychological distress in the Australian National Survey of Mental Health and Well-Being. *Psychological Medicine*, *33*(2), 357–362. https://doi.org/10.1017/S0033291702006700

Gierk, B., Kohlmann, S., Kroenke, K., Spangenberg, L., Zenger, M., Brähler, E., & Löwe, B. (2014). The somatic symptom scale-8 (SSS-8): a brief measure of somatic symptom burden. *JAMA Internal Medicine*, *174*(3), 399–407. https://doi.org/10.1001/jamainternmed.2013.12179

Kessler, R. C., Barker, P. R., Colpe, L. J., Epstein, J. F., Gfroerer, J. C., Hiripi, E., … Zaslavsky, A. M. (2003). Screening for serious mental illness in the general population. *Archives of General Psychiatry*, *60*(2), 184–189. https://doi.org/10.1001/archpsyc.60.2.184

Kessler, R. C., Merikangas, K. R., Berglund, P., Eaton, W. W., Koretz, D. S., & Walters, E. E. (2003). Mild Disorders Should Not Be Eliminated from the DSM-V. *Archives of General Psychiatry*, *60*(11), 1117–1122. https://doi.org/10.1001/archpsyc.60.11.1117

Kroenke, K., Spitzer, R. L., & Williams, J. B. W. (2001). The PHQ-9: Validity of a brief depression severity measure. *Journal of General Internal Medicine*, *16*, 606–613. https://doi.org/10.1046/j.1525-1497.2001.016009606.x

Kurimoto, A., Awata, S., Ohkubo, T., Tsubota-Utsugi, M., Asayama, K., Takahashi, K., … Imai, Y. (2011). Reliability and validity of the Japanese version of the abbreviated Lubben Social Network Scale. *Japanese Journal of Geriatrics*, *48*(2), 149–157. https://doi.org/10.3143/geriatrics.48.149

Lubben, J. E. (1988). Assessing social networks among elderly populations. *Family and Community Health*, *11*(3), 42–52. https://doi.org/10.1097/00003727-198811000-00008

Martire, L. M., Schulz, R., Mittelmark, M. B., & Newsom, J. T. (1999). Stability and change in older adults' social contact and social support: The

Cardiovascular Health Study. *Journals of Gerontology - Series B Psychological Sciences and Social Sciences*, *54B*(5), S302–S311. https://doi.org/10.1093/geronb/54B.5.S302

Matsudaira, K., Kawaguchi, M., Murakami, M., Fukudo, S., Hashizume, M., Oka, H., & Löwe, B. (2016). Development of a Linguistically Validated Japanese Version of the Somatic Symptom Scale-8 (SSS-8). *Japanese Journal of Psychosomatic Medicine*, *56*(9), 931–937. https://doi.org/10.15064/jjpm.56.9_931

Ministry of Health Labour and Welfare. (2020). Comprehensive Survey of Living Conditions. Retrieved May 26, 2020, from https://www.mhlw.go.jp/english/database/db-hss/cslc-index.html

Muramatsu, K, Miyaoka, H., Ueshima, K., Muramatsu, Y., Fuse, K., Yoshimine, H., … Baba, S. (2010). Validation and utility of a Japanese version of the GAD --7. *Japanese Journal of Psychosomatic Medicine*, *50*(6), 166.

Muramatsu, Kumiko, Miyaoka, H., Kamijima, K., Muramatsu, Y., Tanaka, Y., Hosaka, M., … Shimizu, E. (2018). Performance of the Japanese version of the Patient Health Questionnaire-9 (J-PHQ-9) for depression in primary care. *General Hospital Psychiatry*, *52*, 64–69. https://doi.org/10.1016/j.genhosppsych.2018.03.007

Okwumabua, J. O., Baker, F. M., Wong, S. P., & Pilgram, B. O. (1997). Characteristics of depressive symptoms in elderly urban and rural African Americans. *Journals of Gerontology - Series A Biological Sciences and Medical Sciences*, *52*(4), M241–M246. https://doi.org/10.1093/gerona/52A.4.M241

Prochaska, J. J., Sung, H. Y., Max, W., Shi, Y., & Ong, M. (2012). Validity study of the K6 scale as a measure of moderate mental distress based on mental health treatment need and utilization. *International Journal of Methods in Psychiatric Research*, *21*(2), 88–97. https://doi.org/10.1002/mpr.1349

Ruiz, M. A., Zamorano, E., García-Campayo, J., Pardo, A., Freire, O., & Rejas, J. (2011). Validity of the GAD-7 scale as an outcome measure of disability in patients with generalized anxiety disorders in primary care. *Journal of Affective Disorders*, *128*(3), 277–286. https://doi.org/10.1016/j.jad.2010.07.010

Russell, D. W. (1996). UCLA Loneliness Scale (Version 3): Reliability, validity, and factor structure. *Journal of Personality Assessment*, *66*(1), 20–40. https://doi.org/10.1207/s15327752jpa6601_2

Shevlin, M., Murphy, S., & Murphy, J. (2015). The Latent Structure of Loneliness: Testing Competing Factor Models of the UCLA Loneliness Scale in a Large Adolescent Sample. *Assessment*, *22*(2), 208–215. https://doi.org/10.1177/1073191114542596

Siu, A. L., Bibbins-Domingo, K., Grossman, D. C., Baumann, L. C., Davidson, K. W., Ebell, M., … Pignone, M. P. (2016). Screening for depression

in adults: US preventive services task force recommendation statement. *JAMA - Journal of the American Medical Association*, *315*(4), 380–387. https://doi.org/10.1001/jama.2015.18392

Spitzer, R. L., Kroenke, K., Williams, J. B. W., & Löwe, B. (2006). A brief measure for assessing generalized anxiety disorder: the GAD-7. *Archives of Internal Medicine*, *166*(10), 1092–1097. https://doi.org/10.1001/archinte.166.10.1092

Veldhuizen, S., Cairney, J., Kurdyak, P., & Streiner, D. L. (2007). The sensitivity of the K6 as a screen for any disorder in community mental health surveys: A cautionary note. *Canadian Journal of Psychiatry*, *52*(4), 256–259. https://doi.org/10.1177/070674370705200408

Zarei, S., Memari, A. H., Moshayedi, P., & Shayestehfar, M. (2016). Validity and reliability of the UCLA loneliness scale version 3 in Farsi. *Educational Gerontology*, *42*(1), 49–57. https://doi.org/10.1080/03601277.2015.1065688
